# Pulmonary pathology of pandemic influenza A/H1N1 virus (2009)-infected ferrets upon longitudinal evaluation by computed tomography

**DOI:** 10.1099/vir.0.032805-0

**Published:** 2011-08

**Authors:** Edwin J. B. Veldhuis Kroeze, Geert van Amerongen, Marcel L. Dijkshoorn, James H. Simon, Leon de Waal, Ieneke J. C. Hartmann, Gabriel P. Krestin, Thijs Kuiken, Albert D. M. E. Osterhaus, Koert J. Stittelaar

**Affiliations:** 1 ViroClinics BioSciences B.V., 3000 DR Rotterdam, The Netherlands; 2 Department of Virology, Erasmus Medical Center, 3000 DR Rotterdam, The Netherlands; 3 Netherlands Vaccine Institute, 3720 AL Bilthoven, The Netherlands; 4 Department of Radiology, Erasmus Medical Center, 3000 DR Rotterdam, The Netherlands

## Abstract

We investigated the development of pulmonary lesions in ferrets by means of computed tomography (CT) following infection with the 2009 pandemic A/H1N1 influenza virus and compared the scans with gross pathology, histopathology and immunohistochemistry. Ground-glass opacities observed by CT scanning in all infected lungs corresponded to areas of alveolar oedema at necropsy. These areas were most pronounced on day 3 and gradually decreased from days 4 to 7 post-infection. This pilot study shows that the non-invasive imaging procedure allows quantification and characterization of influenza-induced pulmonary lesions in living animals under biosafety level 3 conditions and can thus be used in pre-clinical pharmaceutical efficacy studies.

The ongoing emergence of novel pathogens (CDC, 2009; Osterhaus, 2008) calls for the concomitant development of animal models that address their pathogenesis and assess the potential of preventive and therapeutic intervention strategies. For example, the emergence of the 2009 pandemic A/H1N1 influenza virus (pH1N1) highlighted the need for the rapid development of animal models that closely mimic the human infection (Itoh *et al.*, 2009; Osterhaus, 2008; van den Brand *et al.*, 2010). Studies on the pathogenesis of the disease and the timely assessment of the efficacy and safety of the rapidly developed vaccine candidates, antiviral drugs, antibody preparations and immune modulators, largely depend on newly developed animal models (Del Giudice *et al.*, 2009; Friesen *et al.*, 2010; Itoh *et al.*, 2009; van den Brand *et al.*, 2010; Zhou *et al.*, 2010). However, in these models the assessment of virus-induced lesions is largely based on findings from an arbitrarily chosen time point after experimental infection. When evaluating infections with a peracute onset, significant early findings may be overlooked unless large numbers of animals are sacrificed at consecutive time points. Especially when dealing with outbred animals, like ferrets, evaluation and integration of consecutive findings from different animals may be speculative. In addition, working with highly pathogenic viruses like the pH1N1 virus when it emerged or with the highly pathogenic avian H5N1 influenza viruses, is limited to biosafety level (BSL)-3 laboratory settings. The complexity of working under these stringent restrictions also limits the possibilities to work with large numbers of animals sacrificed at consecutive time points. To overcome these limitations we performed repeated computed tomography (CT) scans of ferrets under BSL-3 conditions before and during infection with the pandemic H1N1 influenza (2009) virus. The pattern of influenza virus attachment and replication in the ferret respiratory tract is largely similar to that in humans (Munster *et al.*, 2009; van Riel *et al.*, 2007), making influenza virus infection of the ferret the model of choice to study human influenza.

The ferrets (*Mustela putorius furo*) used were approximately 8 months of age, females, all seronegative for antibodies against circulating influenza viruses and for antibodies against Aleutian disease virus. They were routinely housed and handled under BSL-3^+^ conditions in negatively pressurized and high efficiency particulate air (HEPA)-filtered biocontainment isolator units, approved by an independent institutional laboratory animal ethics and welfare committee. Animal handling and scans were performed under general injection anaesthesia (ketamine 12.5 mg kg^−1^ and medetomidine-HCl 7.5 µg body weight kg^−1^). Eight ferrets were inoculated intratracheally with 10^6^ TCID_50_ of pandemic influenza virus A/Netherlands/602/2009 (pH1N1) as described previously (Del Giudice *et al.*, 2009; van den Brand *et al.*, 2010). The virus was propagated in Madin–Darby canine kidney cell cultures and the infectious dose was determined as described previously (Munster *et al.*, 2009), and titres calculated according to the method of Spearman–Karber (Kärber, 1931). Virus shedding was monitored daily by collecting nasal and oropharyngeal swabs that were analysed for determination of viral loads by standard procedures (van den Brand *et al.*, 2010), and expressed as log TCID_50_. All animals had detectable levels of virus in their upper respiratory tract (Supplementary Fig. S1, available in JGV Online).

The CT scanner used is a dual-source ultrafast CT system (Somatom Definition Flash; Siemens Healthcare) with a temporal resolution of 0.075 s and table speed of 458 mm s^−1^, the spatial resolution is 0.33 mm. This CT system requires short acquisition times (≈0.22 s) for data recording of an entire ferret thorax. Such a high temporal resolution enables accurate scanning of living ferrets without the necessity of breath holding, respiratory gating or electrocardiogram (ECG) triggering to generate sharp images. During *in vivo* scanning the anaesthetized ferrets were positioned in dorsal recumbency in a perspex biosafety container of approximately 8.3 litre capacity that was purposely designed and built (Tecnilab-BMI) (Supplementary Fig. S2a, b, available in JGV Online). The oxygen concentration in the container did not drop below 14 % as measured by oxymetry. All animals had been scanned 3 days prior to virus inoculation to define the uninfected baseline status of the respiratory system. Four ferrets (#3, #4, #5 and #7) were scanned twice (on days −3 and days 3 or 4), two ferrets (#2 and #8) were scanned three times (on days −3, 4 and 7) and two ferrets (#1 and #6) were scanned four times (on days −3, 3, 4 and 7; Supplementary Table S1, available in JGV Online). In humans, CT-images have been described previously (Gill *et al.*, 2010; Li *et al.*, 2010; Marchiori *et al.*, 2010; Mollura *et al.*, 2009; Perez-Padilla *et al.*, 2009) for pulmonary alterations caused by pandemic (2009) H1N1 influenza virus infection and the histopathological nature of these alterations in humans has been evaluated only to limited extent (Gill *et al.*, 2010; Perez-Padilla *et al.*, 2009). We found consistent bilateral ground-glass opacities in the lungs on all time points of scanning. They were most severe on days 3 and 4 post-infection (p.i.) and showed a reduction on day 7 (Fig. 1). The post-infectious reductions in aerated pulmonary volumes were measured from 3D CT reconstructs using lower and upper thresholds in substance densities of −870 to −430 Hounsfield units (HU). The mean decrease in aerated lung volumes was most pronounced on days 3 (26 cm^3^) and 4 (24 cm^3^) p.i. compared with day 3 before infection. On day 7 the mean aerated lung volume returned to, and equalled, baseline values (31 cm^3^) from day 3 before infection (Table 1).

**Fig. 1.**
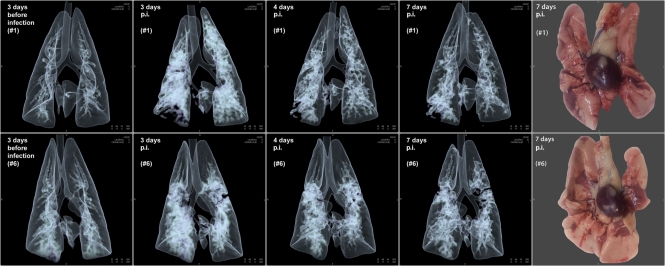
Two rows of four consecutive 3D lung CT images of ferrets #1 and #6 recorded *in vivo* under BSL-3^+^ compared with their appearance at necropsy on the far right. At day 3 before infection, the lungs showed the clear aerated baseline condition, at day 3 p.i. with the new pandemic H1N1 influenza virus marked almost diffuse ground-glass opacities are present that show a gradual reduction towards 7 days p.i. The two photographs taken at necropsy on 7 days p.i. depict the ventral aspect of the lungs, within the centre the hearts still attached to the pulmonary hilus. Both lungs show multifocal reddish consolidated areas of acute inflammation that essentially match with the opacities on the CT images taken just before necropsy; non-affected aerated lung tissue is light pink in colour.

**Table 1.** Pandemic H1N1 (2009)-induced lung lesions as measured by CT (aerated tissue) and histopathology (alveolar oedema and alveolar damage) The aerated lung volumes calculated (used lower and upper thresholds in substance densities: −870 to −430 HU) from the 3D CT reconstructs are presented in cm^3^±sd for all animals individually and averaged on the various days. On 7 days p.i., the mean aerated lung volume returns to, and equals, the mean baseline value on −3 days p.i. of 31 cm^3^. Although not statistically significant, the decrease in lung aeration from baseline value on −3 days p.i. of 31 cm^3^ to 4 days p.i. 24 cm^3^ (*P* = 0.20). The median extent of alveolar damage/alveolitis (score range 0–3) shows a slight decrease from 3 on 4 days p.i. to 2 on 7 days p.i. The median extent of alveolar oedema (score range 0–3) shows a significant (*P* = 0.031) decrease from 3 at 4 days p.i. to 1.5 on 7 days p.i.

	Time (days)	Animal no.								Mean (±SD)	Median (range)
		1	2	3	4	5	6	7	8		
**Mean aerated lung volume (cm^3^)**											
	−3	29±2.2	25±0.4	29±2.7	29±1.8	29±1.7	32±1.2	31±1.9	32±1.2	**31**±4.2†	
	+3	24±3.9	–	–	†*	–	28±1.1	–	–	**26**±3.6	
	+4	37±2.1	17±1.4	31±1.9	–	15±1.5	36±2.7	20±0.2	14±2.2	**24**±9.6	
	+7	48±2.7	11±1.1	–	–	–	33±1.8	–	26±2.3	**31**±14†	
**Alveolar damage (score 0–3)**											
	+4	–	–	3 (2–3)	–	3 (3–3)	–	3 (3–3)	–		**3** (2–3)
	+7	2 (2–3)	2 (2–3)	–	–	–	3 (2–3)	–	2.5 (2–3)		**2** (2–3)
**Alveolar oedema (score 0–3)**											
	+4	–	–	2.5 (2–3)	–	3 (2–3)	–	3 (3–3)	–		**3** (2–3)‡
	+7	1 (1–2)	1.5 (1-2)	–	–	–	2 (1–3)	–	1.5 (1–2)		**1.5** (1–3)‡

* Animal #4 died spontaneously at 3 days p.i.

† Wilcoxon −3 days p.i. versus +4 days p.i., *P* = 0.20.

‡ Mann–Whitney +4 days p.i. versus +7 days p.i., *P* = 0.031.

In addition to CT scanning, we also performed magnetic resonance imaging (MRI) scanning of the ferrets. The MRI scanner used is a High Definition 3 Tesla clinical scanner (General Electric Healthcare) that requires data acquisition times to such a degree that motionless imaging, without the application of respiratory gating and/or ECG triggering, is only possible post-mortem. Because of this limitation and the lower spatial resolution compared with CT scanning, the MRI scan proved impractical for use in this animal model and set-up. Accordingly, the MRI results are not presented. However, MRI scanning under BSL-3 conditions could be of value to image other organ systems *in vivo* that are not hampered by heartbeat or respiratory motion.

Within 1 h after euthanasia by exsanguination from cardiac puncture (time needed for post-mortem MRI scanning) the ferrets were submitted for a full necropsy to compare (histo)pathological data with those that were CT scanned. Animal #4 succumbed spontaneously on day 3 p.i. and had to be necropsied without prior MRI scanning. The entire intact lungs were instilled with, and submerged in 10 % neutral buffered formalin for fixation and disinfection. The lungs were transversely cut just caudal (approx. 10 mm) of the tracheal bifurcation and matched to the same transversal CT image. Additionally from each animal, four similarly cut left lung sections were made not guided by gross lesions. The lung sections were routinely processed, paraffin embedded and 4 µm thin micro-sections were stained with haematoxylin and eosin (H&E) for histopathology. All entire slides were evaluated and scored for the extent of alveolar damage/alveolitis and for the extent of alveolar oedema (0, 0 %; 1, <25 %; 2, 25–50 %; and 3, >50 %). For the detection of influenza A virus-infected cells, additionally serial cut micro-sections were stained for influenza A virus nucleoprotein (NP) as described previously (van Riel *et al.*, 2007).

The pulmonary ground-glass opacities corresponded on histology to extensive alveolar oedema admixed with variable proportions of alveolar macrophages, neutrophils, erythrocytes, fibrin and cellular debris. Immunohistochemical staining for viral NP showed infected pneumocytes lining the inflamed and flooded alveoli (Fig. 2). Additionally, there was a moderate necrotizing bronchiolitis and similar but milder bronchitis. Despite the return to baseline values in mean aerated lung volumes on day 7 p.i. there were still histological lesions mainly in the form of mixed inflammatory cellular infiltrates and type II pneumocyte hyperplasia. Although not statistically significant, the median histopathology scores for the extent of alveolar damage/alveolitis did show a slight decrease from 3 (range 2–3) on day 4 to 2 (range 2–3) on day 7 p.i. Additionally, matching the improvement in lung aeration is a significant (*P* = 0.031) decrease in the median extent of alveolar oedema from 3 (range 2–3) on day 4 to 1.5 (range 1–3) on day 7 p.i. (Table 1).

**Fig. 2.**
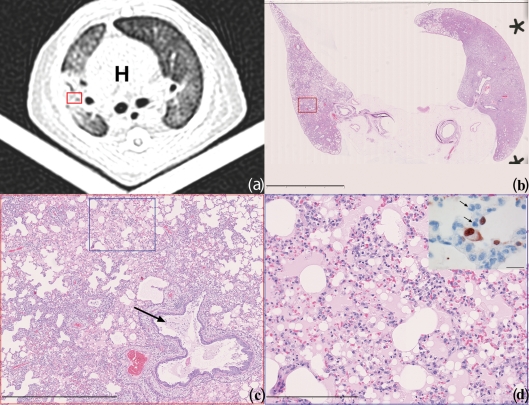
Matching CT scan to histopathology of influenza pH1N1-infected ferret #7 on day 4 p.i. (a) Axial CT-image recorded 10 mm caudally of the tracheal bifurcation depicts bilateral pulmonary ground-glass opacities with peribronchovascular pre-dominance. The rounded white heart shadow (H) is visible in the centre enclosed by the darker lung lobes. During scanning the ferret is placed in dorsal recumbency on a V-shaped tray. The red frame indicates the approximate location of micrograph (c). (b) Subgross histological image matching the location of the axial CT-scan. The red frame indicates the exact location of micrograph (c) (H&E-staining; bar, 10 mm). (c) Low magnification micrograph depicting the histological lesions of the right lung corresponding to consolidated ground-glass opacities, they are composed of extensively flooded alveoli and thickened alveolar septa adjacent to a bronchus containing a plug of mucus with few neutrophils and desquamated epithelial cell remnants (arrow). On top right and middle to bottom left, white non-staining still aerated alveoli are present. The blue frame indicates the exact location of micrograph (d) (H&E-staining; bar, 1 mm). (d) Micrograph depicting the damaged alveoli characterized by infiltrated thickened septa and luminal flooding with protein-rich oedema (pink areas) admixed with variable proportions of macrophages, neutrophils, fibrin, erythrocytes and cellular debris (H&E-staining; bar, 200 µm). Insert on top right: high magnification of serially cut immunohistochemical slide from the same area of micrograph (d), depicting thickened alveolar septa infiltrated by neutrophils (arrows) and adjacent dark-brown staining nuclei of influenza virus-infected pneumocytes lining the flooded alveolus (immunoperoxidase-staining for NP of influenza A virus counterstained with haematoxylin; bar, 40 µm).

We show that monitoring of pulmonary lesions of pH1N1 influenza virus-infected ferrets under BSL-3 conditions by consecutive *in vivo* imaging with CT scanning provides valuable data on disease progression and severity that closely coincide with post-mortem data obtained at the same time points from euthanized animals. The ground-glass opacities observed by CT scanning in all infected lungs largely corresponded to areas of alveolar oedema upon necropsy that were most pronounced on days 3 and 4 and decreased towards day 7. As this method involves repeated CT scans of the same animal instead of sacrificing multiple animals at different time points, it results in a refinement of data collection and a significant reduction in numbers of laboratory animals. In other words the development of respiratory tract lesions of each individual animal can be compared with the situation before infection and followed over time. In this way outbred animals serve as their own baseline control generating more detailed and relevant data per animal. In addition, the assessment of the severity and extent of the lesions over time will lead to more adequate and objective criteria for the time point of euthanasia. The ability of this CT-scanning methodology will not only allow for a more comprehensive study of the pathogenesis of life-threatening infectious diseases, but also of the assessment of the efficacy and safety of vaccination and antiviral strategies against them. In the present pilot study CT scan opacities correspond to alveolar oedema, this parameter is among others used as read out in influenza vaccine efficacy studies (Baras *et al.*, 2011; van den Brand *et al.*, 2011). Obviously this methodology is not limited to studying the respiratory tract, but could also be exploited for new emerging pathogens with their specific target organs in other animal models.

## References

[r1] Baras B. de Waal L. Stittelaar K. J. Jacob V. Giannini S. Veldhuis Kroeze E. J. van den Brand J. M. A. van Amerongen G. Simon J. H. **(**2011**).** Pandemic H1N1 vaccine requires the use of an adjuvant to protect against challenge in naïve ferrets. Vaccine 29, 2120–2126 10.1016/j.vaccine.2010.12.125 21238573

[r2] CDC **(**2009**).** Update: infections with a swine-origin influenza A (H1N1) virus–United States and other countries, April 28, 2009. MMWR Morb Mortal Wkly Rep 58, 431–433 19407737

[r3] Del Giudice G. Stittelaar K. J. van Amerongen G. Simon J. Osterhaus A. D. Stöhr K. Rappuoli R. **(**2009**).** Seasonal influenza vaccine provides priming for A/H1N1 immunization. Sci Transl Med 1, re1 20371459 10.1126/scitranslmed.3000564

[r4] Friesen R. H. Koudstaal W. Koldijk M. H. Weverling G. J. Brakenhoff J. P. Lenting P. J. Stittelaar K. J. Osterhaus A. D. Kompier R. Goudsmit J. **(**2010**).** New class of monoclonal antibodies against severe influenza: prophylactic and therapeutic efficacy in ferrets. PLoS ONE 5, e9106 10.1371/journal.pone.0009106 20161706 PMC2817000

[r5] Gill J. R. Sheng Z. M. Ely S. F. Guinee D. G. Beasley M. B. Suh J. Deshpande C. Mollura D. J. Morens D. M. **(**2010**).** Pulmonary pathologic findings of fatal 2009 pandemic influenza A/H1N1 viral infections. Arch Pathol Lab Med 134, 235–243 20121613 10.5858/134.2.235 PMC2819217

[r6] Itoh Y. Shinya K. Kiso M. Watanabe T. Sakoda Y. Hatta M. Muramoto Y. Tamura D. Sakai-Tagawa Y. **(**2009**).** *In vitro* and *in vivo* characterization of new swine-origin H1N1 influenza viruses. Nature 460, 1021–1025 19672242 10.1038/nature08260 PMC2748827

[r7] Kärber G. **(**1931**).** Beitrag zur kollektiven Behandlung pharmakologischer Reihenversuche. Arch Exp Path Pharmak 162, 480–483 (in German). 10.1007/BF01863914

[r8] Li P. Su D. J. Zhang J. F. Xia X. D. Sui H. Zhao D. H. **(**2010**).** Pneumonia in novel swine-origin influenza A (H1N1) virus infection: high-resolution CT findings. Eur J Radiol. 10.1016/j.ejrad.2010.05.029 20566254 PMC7185744

[r9] Marchiori E. Zanetti G. Hochhegger B. Rodrigues R. S. Fontes C. A. Nobre L. F. Mançano A. D. Meirelles G. Irion K. L. **(**2010**).** High-resolution computed tomography findings from adult patients with influenza A (H1N1) virus-associated pneumonia. Eur J Radiol 74, 93–98 10.1016/j.ejrad.2009.11.005 19962842

[r10] Mollura D. J. Asnis D. S. Crupi R. S. Conetta R. Feigin D. S. Bray M. Taubenberger J. K. Bluemke D. A. **(**2009**).** Imaging findings in a fatal case of pandemic swine-origin influenza A (H1N1). AJR Am J Roentgenol 193, 1500–1503 10.2214/AJR.09.3365 19933640 PMC2788497

[r11] Munster V. J. de Wit E. van den Brand J. M. Herfst S. Schrauwen E. J. Bestebroer T. M. van de Vijver D. Boucher C. A. Koopmans M. **(**2009**).** Pathogenesis and transmission of swine-origin 2009 A(H1N1) influenza virus in ferrets. Science 325, 481–483 19574348 10.1126/science.1177127 PMC4814155

[r12] Osterhaus A. D. **(**2008**).** New respiratory viruses of humans. Pediatr Infect Dis J 27 Suppl. S71–S74 10.1097/INF.0b013e3181684d7c 18820582

[r13] Perez-Padilla R. de la Rosa-Zamboni D. Ponce de Leon S. Hernandez M. Quiñones-Falconi F. Bautista E. Ramirez-Venegas A. Rojas-Serrano J. Ormsby C. E. **(**2009**).** Pneumonia and respiratory failure from swine-origin influenza A (H1N1) in Mexico. N Engl J Med 361, 680–689 10.1056/NEJMoa0904252 19564631

[r14] van den Brand J. M. Stittelaar K. J. van Amerongen G. Rimmelzwaan G. F. Simon J. de Wit E. Munster V. Bestebroer T. Fouchier R. A. **(**2010**).** Severity of pneumonia due to new H1N1 influenza virus in ferrets is intermediate between that due to seasonal H1N1 virus and highly pathogenic avian influenza H5N1 virus. J Infect Dis 201, 993–999 10.1086/651132 20187747 PMC7110095

[r15] van den Brand J. M. Kreijtz J. H. Bodewes R. Stittelaar K. J. van Amerongen G. Kuiken T. Simon J. Fouchier R. A. Del Giudice G. **(**2011**).** Efficacy of vaccination with different combinations of MF59-adjuvanted and nonadjuvanted seasonal and pandemic influenza vaccines against pandemic H1N1 (2009) influenza virus infection in ferrets. J Virol 85, 2851–2858 10.1128/JVI.01939-10 21209108 PMC3067945

[r16] van Riel D. Munster V. J. de Wit E. Rimmelzwaan G. F. Fouchier R. A. Osterhaus A. D. Kuiken T. **(**2007**).** Human and avian influenza viruses target different cells in the lower respiratory tract of humans and other mammals. Am J Pathol 171, 1215–1223 10.2353/ajpath.2007.070248 17717141 PMC1988871

[r17] Zhou B. Li Y. Belser J. A. Pearce M. B. Schmolke M. Subba A. X. Shi Z. Zaki S. R. Blau D. M. García-Sastre A. **(**2010**).** NS-based live attenuated H1N1 pandemic vaccines protect mice and ferrets. Vaccine 28, 8015–8025 10.1016/j.vaccine.2010.08.106 20934458 PMC2991506

